# Simultaneous progression patterns of scoliosis, pelvic obliquity, and hip subluxation/dislocation in non-ambulatory neuromuscular patients: an approach to deformity documentation

**DOI:** 10.1007/s11832-015-0683-7

**Published:** 2015-09-30

**Authors:** Janki Patel, Frederic Shapiro

**Affiliations:** St. Louis, MO USA; Boston Children’s Hospital, Boston, MA USA

**Keywords:** Scoliosis, Pelvic obliquity, Hip subluxation/dislocation, Deformity documentation

## Abstract

**Background:**

A triad of deformities—thoracolumbar scoliosis, pelvic obliquity, and femoral head (hip) subluxation/dislocation—occurs frequently in non-ambulatory neuromuscular patients, but their close inter-relationship is infrequently appreciated or quantified. We propose a deformity documentation approach to assess each component simultaneously.

**Methods:**

The documentation assesses each component for maximal functional level, deformity, and flexibility/rigidity: deformity from antero-posterior radiographs (scoliosis—maximal functional position, pelvic obliquity—sitting, hip position—supine) and flexibility/rigidity from extent of repositioning on supine (spine, pelvis) and frog lateral (hip) radiographs. The approach was applied in 211 patients: Duchenne muscular dystrophy (110), spinal muscular atrophy (49), cerebral palsy (26), and other neuromuscular disorders (26).

**Results:**

Measurement of 2124 radiological data points allowed for deformity (mild to moderate to severe) and flexibility/rigidity (fully reducible to partially to non-reducible) gradations for scoliosis, pelvic obliquity, and hip subluxation/dislocation. The charting documented: (1) numerical deformity and flexibility/rigidity changes [*x*-axis: age; *y*-axis: angulation (scoliosis and pelvic obliquity) and percent coverage (hip subluxation or dislocation) from 0–120]; and (2) grade deformity and flexibility/rigidity changes [*x*-axis: age; *y*-axis: deformity and flexibility/rigidity, following conversion of numerical measurements to a 1–5 grade scale]. In subgroups with the most extensive documentation, thoracolumbar and lumbar scoliosis extended into the sacrum with 98 % (114/116) accompanied by pelvic obliquity; and scoliosis developed more rapidly than hip deformity in 44 % (28/63), scoliosis and hip deformity developed at the same time in 40 % (25/63), and hip deformity developed more rapidly than scoliosis in 16 % (10/63) (Pearson’s chi-squared test *p* = 0.0501, almost significant).

**Conclusion and significance:**

Documentation of the triad of neuromuscular deformities is applicable to all diagnoses; it outlines maximal functional level, deformity, and flexibility/rigidity at each site; and it shows the relationship between spine, pelvic, and hip deformation. Prospective charting will enhance both clinical management and clinical research into neuromuscular deformity.

## Introduction

A triad of deformities—thoracolumbar scoliosis, pelvic obliquity, and femoral head (hip) subluxation/dislocation—occurs frequently in non-ambulatory neuromuscular patients, and their close inter-relationship warrants continuing analysis. While there has been recognition by several observers of the relationship of these deformities to one another and of their tendency to worsen with time [1–8], we continue to see patients developing severe deformity in the first and early second decades of life with orthotics, wheelchair modification, and surgical management performed late, only after severe and rigid deformity has occurred. We report a study of deformity progression leading to a documentation approach involving each component of the deformity complex. By describing and charting the inter-related disorders simultaneously the greater awareness provided should lead to earlier, more effective, and less complex treatment.

## Methods

### Components assessed

**Fig. 1:**
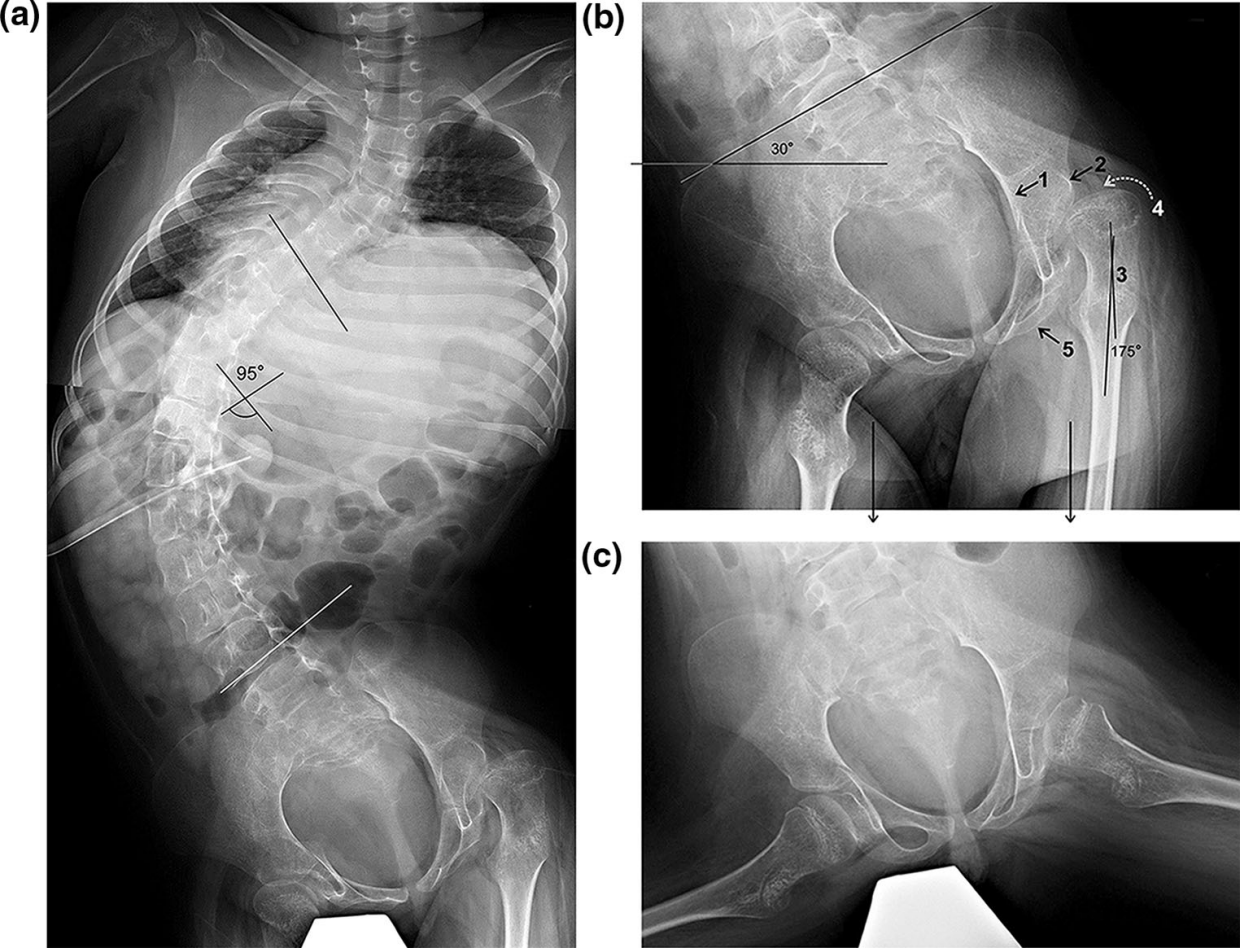
Radiological images outline the inter-related spinal, pelvic, and hip deformities in a patient with severe quadriparetic hypotonic cerebral palsy. **a** The classic fully developed triadic deformity is illustrated. The thoracolumbar scoliosis measures 95°, there is marked pelvic obliquity, and the left hip is severely subluxed. **b** Antero-posterior radiograph centered on the pelvis shows the severe lumbosacral scoliosis continuing into the pelvis, marked pelvic obliquity, and severe subluxation of the left femoral head with coxa valga. This radiographic projection is taken after specific positioning of the patient for assessment of the flexibility component of pelvic obliquity. The patient lies supine on the radiological table with both femurs (two longitudinal axis *arrows* at *bottom* of image) parallel (as though the patient was standing upright) to allow for pelvic obliquity measurements. An *oblique line* connects the most superior parts of the two iliac crests and the transverse line through the pelvis is at right angles to the two sides of the radiological image (or to the parallel lines of the properly positioned femurs). The rigid pelvic obliquity measures 30°. The left hip is severely subluxed in association with the pelvic obliquity. *Numbers and measurements* at the *left* hip region indicate contributing pathogenesis of hip displacement in the triad of deformities: *1* acetabular tilt away from femoral head; *2* under-development of lateral acetabular cartilage and bone; *3* proximal femoral coxa valga with the head-neck/shaft angle 175°; *4* proximal femoral anteversion (indicated by *dotted arrow*); and *5* adductor muscle tightness. **c** Example of full hip flexibility is seen on the frog lateral radiograph showing complete relocation of the severely subluxed femoral head pictured above in Fig. 1a, b

Each component of the deformity triad was assessed in terms of the maximal functional level for the patient (supine, sitting, walking), the degree of deformity at each site, and the flexibility/rigidity of each deformity. Deformity measurements were made for scoliosis, from antero-posterior frontal (coronal) plane spine radiographs in the maximal functional position; for pelvic obliquity, from sitting antero-posterior spine radiographs that included the proximal levels of the sacrum and both iliac crests; and for hip position, from supine antero-posterior bilateral hip radiographs. Flexibility/rigidity determinations were based on the extent of repositioning from the deformity measurements of the above-listed radiographs as defined by, for scoliosis, antero-posterior spine radiographs in supine, supine bending or seated in brace positions; for pelvic obliquity, supine antero-posterior pelvic radiographs with specific positioning; and for hip position, bilateral frog lateral hip radiographs. The radiographic views are outlined in Fig. 1.

### Deformity assessments

#### Spine

Antero-posterior spine radiographs were measured using the Cobb criteria. Normal spine measurement in assessing for scoliosis using standing, sitting, or supine radiographs for the purpose of this study was considered to be 0° since in this patient population slight deformity beyond 0° generally indicates the beginning of the progression into scoliosis. In the large majority of scoliosis cases, the lumbar component of the curve continued beyond L5 into the sacrum, stressing the need for spinal radiographs to include the lumbosacral junction.

#### Pelvis

Pelvic obliquity was measured on sitting antero-posterior full spine radiographs. A line was drawn linking the most superior part of each (right and left) iliac crest, the horizontal axis (in the sitting position) was parallel to the lowermost exposure line of the radiograph at the lower end of the pelvis, and pelvic obliquity was the angle formed by these two lines. While the optimal measurement is 0°, we consider any measurement in the range of 0–1.9° to be normal since values of zero are not invariably seen and, in a practical sense, there is often difficulty in measuring in this patient population.

#### Hip

Hip position was quantified by determining the migration percentage assessing the ossified femoral head coverage by the bony acetabulum. Hip subluxation was measured on the antero-posterior bilateral hip radiograph by drawing a perpendicular line (to the horizontal axis) from the outer bony margin of the acetabulum (Perkin’s line) passing distally through the femoral head. The migration percentage (or index) is a measure of the percentage of the ossified femoral head left uncovered by the ossified acetabular roof; that is, the amount (or percentage) of femoral head outside (lateral to) Perkins’ line on the frontal view [9–11]. This measurement also indicates the percentage of the femoral head covered by the acetabulum and this latter parameter was used to quantify position. Normal coverage of each femoral head by the outer edge of the acetabulum is considered to be 67 % or greater on the antero-posterior radiograph (2/3 of the bone of the femoral head is contained within the lateral bony margin of the acetabulum, with 1/3 outside).

### Flexibility/rigidity assessments

Flexibility was measured in the supine position for each of the three regions. This position effectively removes the deforming forces of gravity and weakness present in the upright position. Measurements of spinal, pelvic, and hip flexibility led to gradations from normal (full correction) to a series of intermediate values indicating partial correction of deformity with some flexibility to being unchanged from the deformity group (severe rigidity). Flexibility and rigidity thus occur at opposite ends of the same spectrum.

#### Spine

Spinal flexibility which decreases scoliosis deformity can be determined in three ways: (1) antero-posterior spine radiograph in the supine position (out of brace); (2) antero-posterior spine radiograph in the supine position with bending to straighten the spine (i.e., bending towards the convexity and away from the concavity); and (3) antero-posterior spine radiograph in the sitting position in brace. The Cobb angle is then measured and compared with the scoliosis deformity angle in the maximal functional position out of brace.

#### Pelvis

Pelvic obliquity can often be seen to diminish in sitting antero-posterior spine radiographs in brace, indicating some degree of flexibility in pelvic deformity. It became evident by qualitative observation of large numbers of radiographs that pelvic obliquity also tends to diminish in supine compared to sitting radiographs, at least until rigidity sets in. There is, however, no standardized way to document this. We developed a way to document supine pelvic obliquity (and thus flexibility of the pelvic obliquity deformity) by carefully positioning the patient supine on the radiographic table with the head, trunk, and lower extremities as anatomically straight as possible. The patient was positioned with each segment (trunk, pelvis, lower extremities) centered as closely as possible in relation to either side of the table. The radiograph was centered on the pelvis but included the lumbosacral region of the spine and the proximal half of both femurs. To determine supine pelvic obliquity, the horizontal axis for measurement is a line placed within the pelvis perpendicular to left and right sides of the image and the iliac crest axis is the line linking the most superior point of each iliac crest. The angle formed between these two lines is the supine pelvic obliquity.

#### Hip

The frog lateral hip radiograph determines the extent of hip deformity flexibility. A dislocated or subluxed femoral head on the antero-posterior view can either reduce fully into the acetabulum, partially, or not at all. The migration percentage can be determined on the frog lateral radiograph by the same method of measurement used on the frontal antero-posterior view since the pelvis and acetabulae are in the same position for both projections and it is only the proximal femurs that move with the frog lateral positioning.

**Fig. 2:**
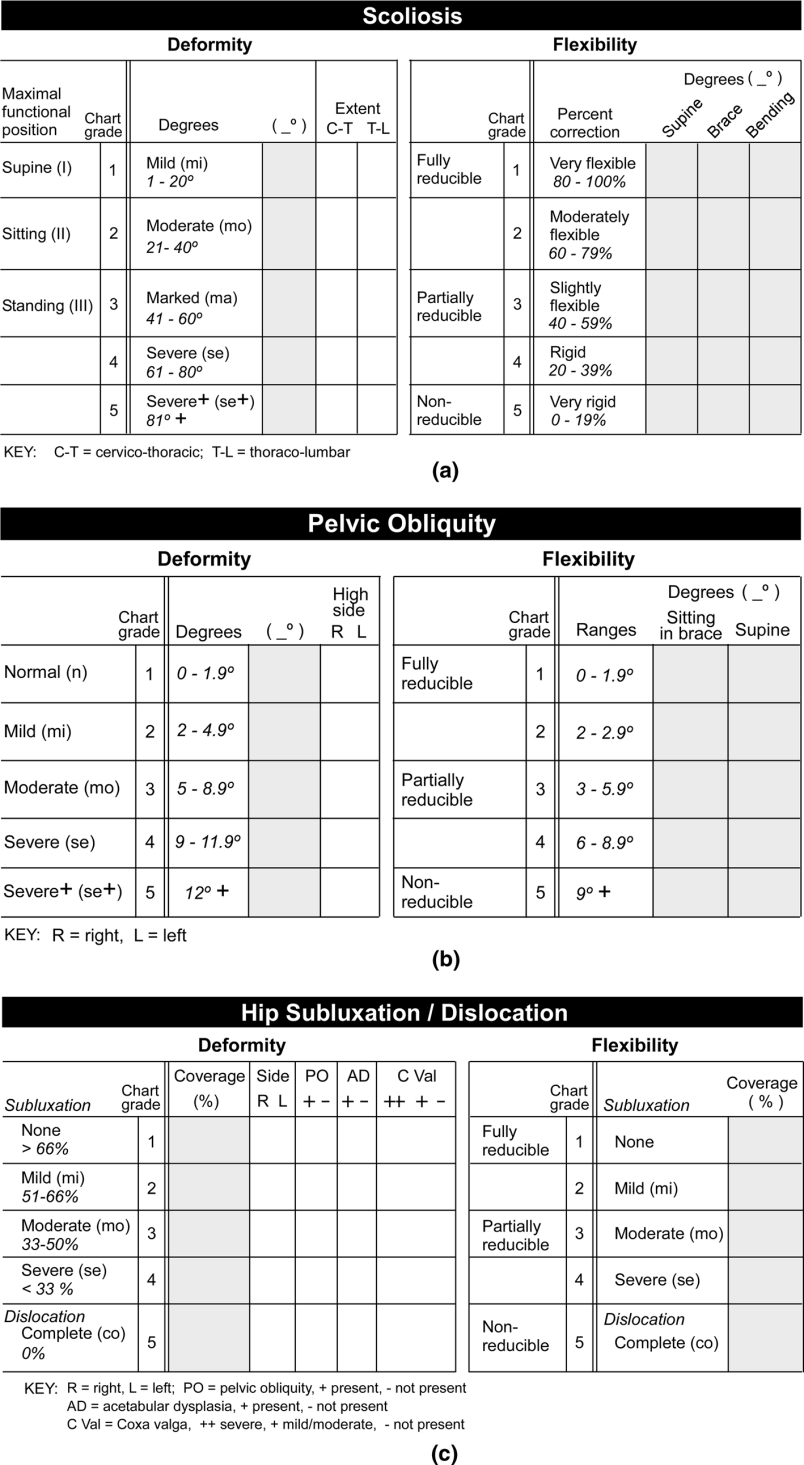
The tabular format for documenting each of the three components of the inter-related deformities. **a** Scoliosis documentation in tabular format. **b** Pelvic obliquity documentation in tabular format. **c** Hip subluxation/dislocation documentation in tabular format

The deformity and flexibility assessments are outlined in Table 1 and Fig. 2a–c.

**Table 1:**
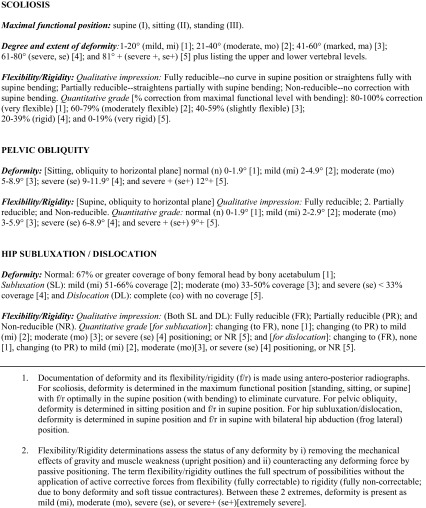
Parameters for deformity and flexibility/rigidity documentation^1,2^

### Chart method

**Fig. 3:**
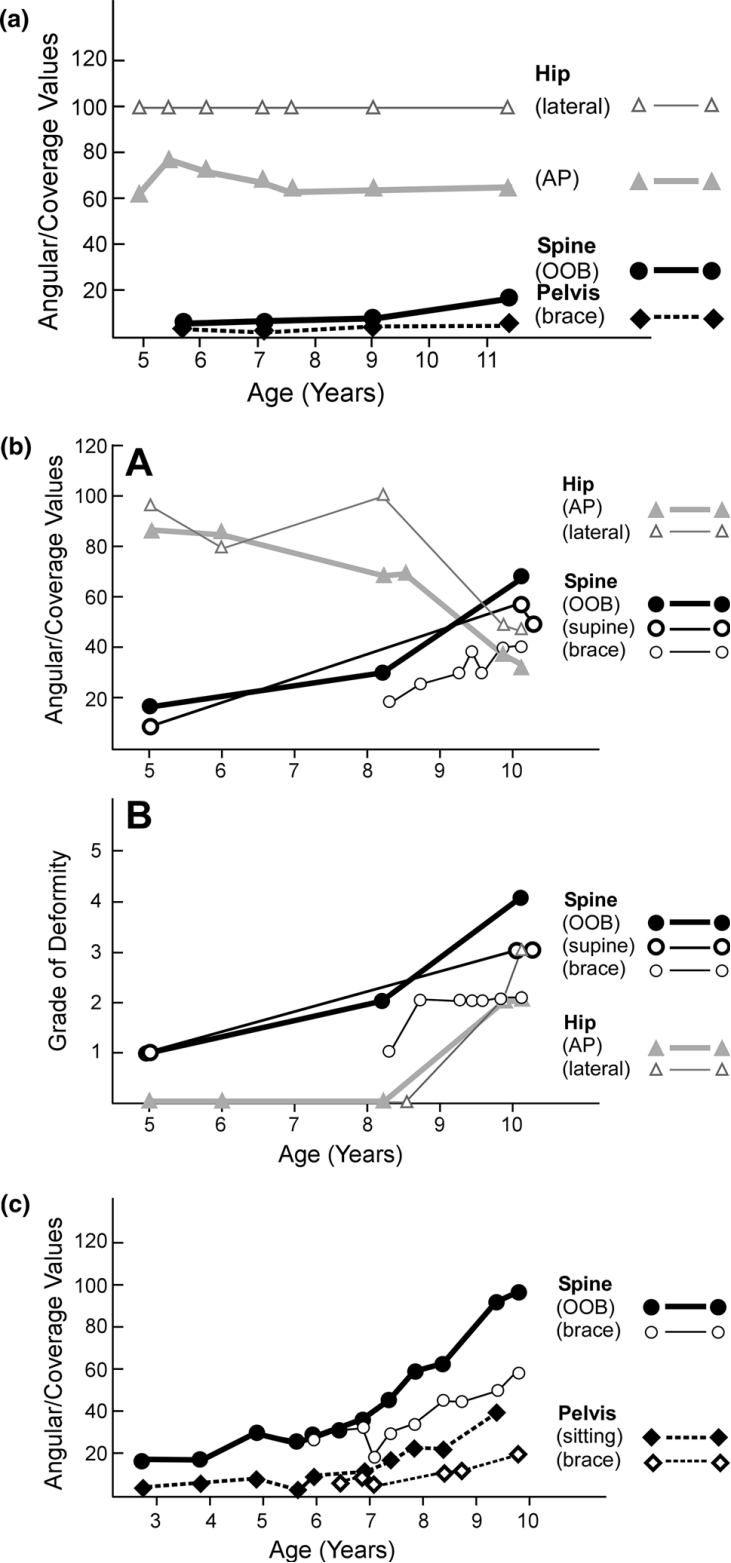

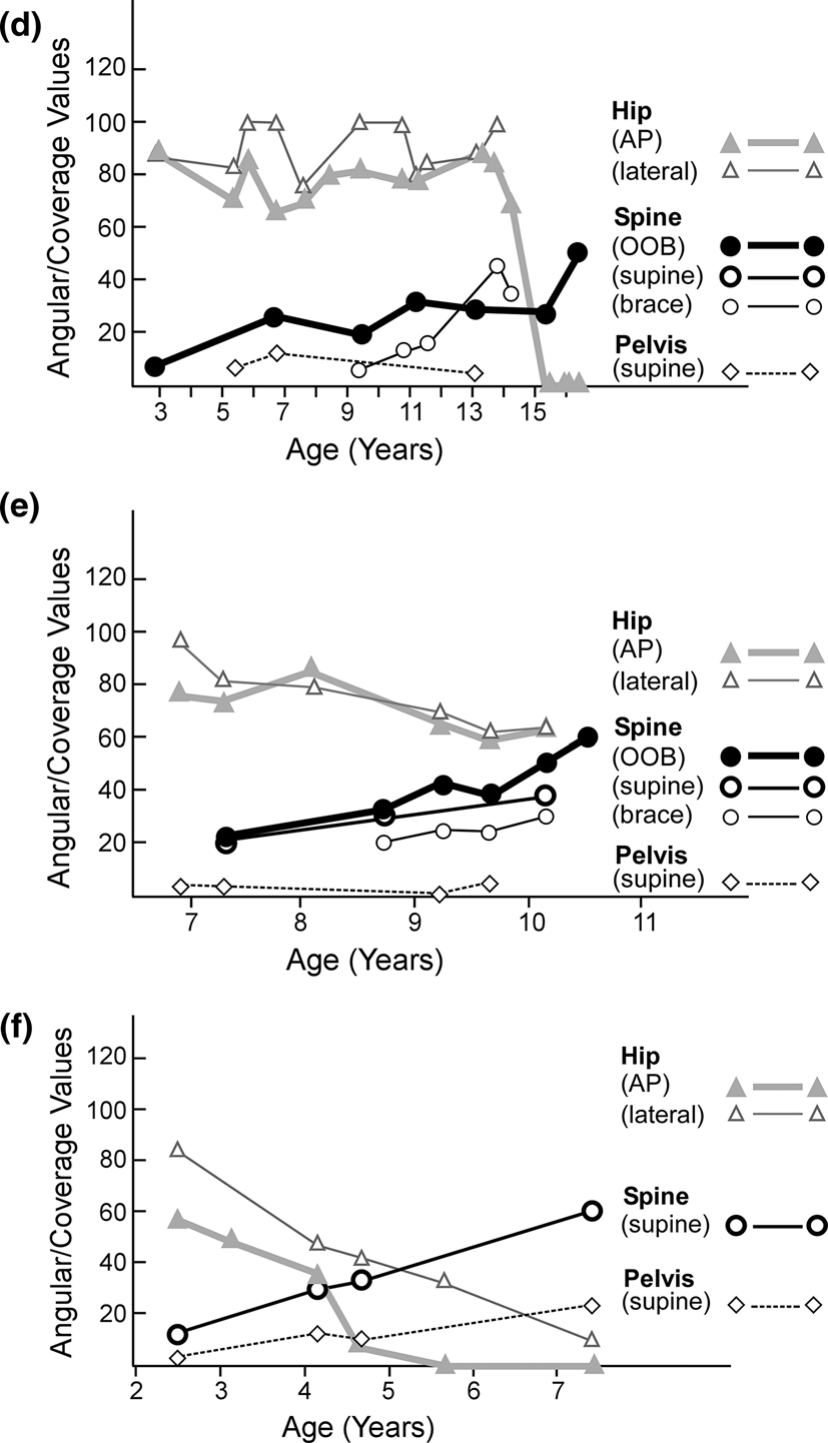
Examples of numerical and grade charting for deformity assessments. Age in years is marked along the *x*-*axis*. In the numerical deformity and flexibility/rigidity charts the *y*-*axis* *numbers* 0–120 indicate degrees (angular values) for scoliosis and pelvic obliquity and percent coverage of the femoral head by the acetabulum for hip position. In the grade deformity and flexibility/rigidity chart the *y*-*axis* *numbers* 1–5 indicate the quantitative grades corresponding to the numerical values measured as outlined in Fig. 2a, b, c. The same code is used from chart to chart. Spine (OOB) refers to a sitting antero-posterior spine radiograph out of brace. Spine (supine) is an antero-posterior spine radiograph supine to straighten the curve passively. Not shown in these figures is Spine (bending) that refers to a supine bending spine radiograph to further straighten the spine. Spine (brace) refers to a sitting antero-posterior spine radiograph in brace. Pelvis (sitting) refers to a sitting antero-posterior spine radiograph visualizing the iliac crests to assess for pelvic obliquity. Pelvis (brace) refers to a sitting antero-posterior spine radiograph visualizing the iliac crests in brace. Pelvis (supine) refers to an antero-posterior pelvis radiograph in balanced position supine (trunk, pelvis, lower extremities positioned centrally on table with femurs parallel to long axis of table) to assess flexibility of any pelvic obliquity. Hip (AP) refers to a bilateral antero-posterior hip radiograph in the supine position that defines deformity. Hip (lateral) refers to a bilateral frog lateral hip radiograph in supine position that defines flexibility/rigidity. **a** Numerical values in a mildly involved patient with spinal muscular atrophy type III show an almost normal profile with no hip displacement, no pelvic obliquity, and a scoliosis measuring only 10°. **b** Numerical deformity values (*top* *A*) are shown for a patient with severe quadriparetic spastic cerebral palsy. Deformities of spine and hip are documented. Spinal values of deformity increased from 5 to 10 years of age with sitting out of brace (OOB) deformity greatest, supine less, and deformity in brace least. Corresponding grade deformity values (*bottom B*) are shown. The main differences in numerical and grade chartings relate to hip deformity with numerical values (*top* *chart*) decreasing dramatically with worsening subluxation to dislocation while grade representations (*lower chart*) increase with dislocation since severity grading (towards 5) slants upwards. **c** Numerical deformity values are shown for a patient with spinal muscular atrophy type II. Deformities of spine and pelvis are shown. Spinal deformity sitting out of brace (OOB) worsens to 100° but bracing diminishes curve to 55°. Note pelvic obliquity sitting without support. Pelvic obliquity is depicted at 40° sitting out of brace but decreased to 20° sitting in brace. **d** Numerical deformity values are shown for a patient with severe quadriparetic spastic cerebral palsy. Deformities of spine, pelvis, and hip are documented with good separation and visualization on the single chart. The rapid downward slope represents hip dislocation over a period of time from 13 to 15 years of age. Note that the hip AP view at 15 years of age indicates complete dislocation while, at the same time, the lateral hip view indicated excellent flexibility with complete relocation and coverage documented. **e** Numerical deformity values are shown for a patient with severe quadriparetic spastic cerebral palsy. Deformities of hip, spine, and pelvis are documented. **f** Numerical deformity values are shown for a patient with spinal muscular atrophy type II. Deformity in each of the 3 regions is advancing rapidly. The hip is dislocated at 5 years of age (hip AP) and irreducible on the frog lateral view at 7 years of age (hip lateral). Scoliosis progressively increases and is 60° at 7 years of age while pelvic obliquity is 20° at 7 years of age

To document deformity values in relation to time, two methods were used (Fig. 3a–f). The first plots the numerical deformity values for scoliosis, pelvic obliquity, and hip position as well as the corresponding supine flexibility/rigidity measurements. The second converts these numerical measurements into quantitative grades 1–5 and plots the grades.

#### Numerical deformity and flexibility/rigidity values

Each deformity is listed using a specific symbol and line linking each study date. Specific symbols along with associated specific shadings (black, gray, white), line thickness, and line pattern (solid, dotted, etc.) were established for sitting AP (antero-posterior) spine out of brace (OOB), sitting AP spine in brace (brace), supine AP spine (supine), and supine bending spine (bending); AP pelvis sitting (sitting), AP pelvis sitting in brace (brace) and pelvis supine (supine); and hip AP (AP) and hip frog lateral (lateral). The age of the patient at the time of each study is shown along the horizontal *x*-axis while the vertical *y*-axis numbered from 0 to 120 indicates both deformity and flexibility/rigidity by angular measurements in degrees for scoliosis and pelvic obliquity and the percent coverage of the femoral head by the acetabulum for hip position. Considering the ranges of scoliosis, pelvic obliquity, and femoral head coverage, the values tend to be dispersed at specific regions of the chart in the normal or with early deformity, allowing simultaneous visual appreciation of the pattern of positioning with time. In a normal child or one with very little deformity, the percent coverage of the femoral head is towards the upper part of the 0–120 range while the scoliosis and pelvic obliquity readings fall in the bottom part. With this method of numerical charting, as scoliosis and pelvic obliquity increase (worsen) the values track upwards; as hip position progressively worsens towards full dislocation, however, the percent coverage decreases and a downward slope for the values indicates progressive deformity.

#### Grade deformity and flexibility/rigidity values

As increasing numbers of patients were assessed, the specific numerical ranges for grading each deformity and its degree of flexibility/rigidity from mild to moderate to severe allowed for an alternative way of tracking deformation. In a qualitative sense these changes are referred to as fully reducible, partially reducible, and non-reducible, although these concepts are not charted. For charting, age was plotted in similar fashion on the *x*-axis, with grades from 1 (least deformity) to 5 (greatest deformity) placed along the *y*-axis. The grade correspondences to the numerical ranges of deformity and flexibility/rigidity for each of the three regions are shown in Fig. 2a–c. With this method of charting by grade, worsening in each of the three parameters of deformity always tracks upwards.

### Patient studies

Inclusion in the study was based on retrospective review of all radiographs in patients with severe non-ambulatory neuromuscular disorders who had been followed by the senior author (FS) over a period of several years in the neuromuscular clinic and a smaller cerebral palsy clinic. Radiographs of the three regions being assessed for triadic deformities were excluded if they did not clearly display each region or if they were not taken simultaneously, by which is meant (in the context of this study) each region on the same day or, if not possible, within a few days of each other. Assessments were concentrated in DMD patients after they became wheelchair-dependent; SMA patients who had type II (all non-ambulatory) [with only a few type I (either supine or semi-sitting as their maximal functional level) and type III (who became non-ambulatory towards the end of the first decade)]; and CP patients with GMFCS (gross motor function classification system) levels IV and V [12] with severe non-ambulatory quadriparesis. Each radiograph (spine, pelvis, hip) for the study was measured and documented. To be included in this retrospective study, measurements at any time period required both deformity and flexibility/rigidity components. The deformity and flexibility/rigidity determinations were measured in degrees for scoliosis and pelvic obliquity and as percent coverage of the femoral head for hip subluxation/dislocation. Full flexibility allowed for complete correction of deformity and at the other end of the spectrum rigid deformities remained non-reducible, explaining the use of the term flexibility/rigidity. Between the two extremes, deformities that were partially corrected were listed as mild (mi), moderate (mo), severe (se) or very severe (severe +).

Documentation was applied for validation of this approach in 211 patients with neuromuscular disorders. The large majority of assessments were done in patients with severe involvement in the non-ambulatory phase of the disorder. At this stage of weakness and/or spasticity, the triad of deformities occurs and worsens with time as the clinical conditions worsen. Serial data points from each patient were charted to assess the development and progression of each deformity, its flexibility/rigidity, and their inter-relationships. Diagnoses and numbers of patients comprised Duchenne muscular dystrophy (DMD) 110, spinal muscular atrophy (SMA) 49, cerebral palsy (CP) 26, and other neuromuscular disorders [including limb girdle muscular dystrophy (LGMD), myopathy, and neurodegenerative disorders] 26. Radiographic measurements totaled 2124 data points.

Data were accumulated to establish the numerical ranges of scoliosis, pelvic obliquity, and hip subluxation/dislocation ranging from normal values through the range of mild, moderate, severe, and severe plus levels of deformation. Relative patterns of progression were also outlined based on the frequency of radiological studies in the following distributions: a large number of radiographs were available assessing each of the three regions (spine, pelvis, hip) for both deformity and flexibility on three or more occasions at least 1 year apart; some radiographs were available assessing spine and hip in the same time frame on two occasions at least 1 year apart; or only on one occasion. A set of studies refers to spine, pelvis, and hip or spine and hip radiographs taken in the same time frame. The same time frame refers to radiographs of the three regions taken preferably on the same day or, if not possible, within a few days of each other.

As well as determining related patterns of deformity progression at each site, the highest quality studies in the largest subset of patients (assessing spine, pelvis, and hips on 3 or more occasions at least 1 year apart) (*N* = 63) determined whether spine and hip deformities progressed at an equal rate, with spine deformation more rapid than hip deformation or with hip deformation more rapid than spine deformation. The findings were assessed statistically using Pearson’s chi-squared test to determine the *p* value. Another large subset (with radiographs clearly showing the lumbar spine, both iliac crests, and the sacrum close to skeletal maturity) (*N* = 116) assessed the relationship between the lumbar or lumbosacral scoliosis and pelvic obliquity. Study distributions for the 211 patients listed (in order) as three or more sets of studies, two sets of studies, or one set of studies are total (211): 79, 77, 55; DMD (110): 12, 53, 45; SMA (49): 35, 9, 5; CP (26): 18, 7, 1; and other NM (26): 14, 8, 4.

## Results

### Radiographic examples

Figure 1a illustrates a classic triadic deformity with a prominent thoracolumbar scoliosis curve continuous into the sacrum, pelvic obliquity, and hip subluxation on the high side of the pelvis at the concave side of the thoracolumbar curve. Figures 1b and 1c highlight specific regions of deformity. Full hip flexibility is illustrated on the frog lateral radiograph as the dislocated femoral head repositions completely into the acetabulum (Fig. 1c).

### Deformity and flexibility assessments

Deformity and flexibility/rigidity assessments, determined from the numerous values from the radiological imaging, are detailed in Table 1 and Fig. 2a–c.

### Patterns of inter-related development

Three patterns of inter-related deformity were identified in a subset of 63 patients who had the most frequent documentation (3 or more sets of high-quality radiographs taken in the same time frame and allowing for measurement of spine and hip deformity) using the grade charting values. The patterns were development of spine deformity more rapidly than hip deformity, development of spine and hip deformity at the same time, or development of hip deformity more rapidly than spine deformity (Table 2). When assessed statistically using Pearson’s chi-squared test the *p*-value is almost significant (*p* = 0.0501) using a significance level of <0.05. Spine and hip deformities developed at the same time in 25/63 (40 %) of patients but spine deformities developed more rapidly than hip in 28/63 (44 %) while hip deformities developed more rapidly than spine deformities in only 10/63 (16 %). Seven of the 10 patients who developed hip deformities first were in the cerebral palsy group.

**Table 2:** Patterns of inter-related deformity development in non-ambulatory neuromuscular patients

	Spine deformities develop more rapidly than hip deformities	Spine and hip deformities develop at same time	Hip deformities develop more rapidly than spine deformities
Total	28 (44.4 %)	25 (39.7 %)	10 (15.9 %)
SMA	10	15	1
CP	11	8	7
DMD	7	2	2

Based on 63 best-documented patients. Pearson’s chi-squared test *df* = 4, *χ* ^2^ = 9.48, *p*-value = 0.050136 (almost significant)

In another subset of 116 well-documented patients, 114/116 (98 %) with a lumbar or thoracolumbar scoliosis were associated with pelvic obliquity greater than 2° (with 0°–1.9° defined as normal in this study). Both patients with normal pelvic obliquity had DMD, one with only a 5° curve but the other with a 60° deformity.

### Progression pattern charting

Detailed charts illustrate the information available from this approach following patients with the three inter-related deformities (Fig. 3a–f). Figure 3a shows the numerical chart from a patient with mild spinal muscular atrophy III that essentially represents the normal appearance since only a minimal scoliosis of 10° was present at the most recent assessment. Corresponding numerical and grade deformity charts are shown for a patient with severe spastic quadriparetic cerebral palsy (Fig. 3b). Numerical deformity charts alone are shown for other patients in Fig. 3c–f.

### Supine pelvic obliquity measurements

This study describes our method for measuring pelvic obliquity in the supine position to help demonstrate its flexibility. As our experience with this technique evolved, we compared the sitting and supine positions where available. The supine values were less than the sitting values, being approximately one-half of the sitting values with mild deformity and increasing as deformity worsened to 2/3 (with grade 3) and to 3/4 (with grades 4–5) of the sitting values.

## Discussion

### Simultaneous deformity and flexibility/rigidity documentation

The deformity documentation approach shows the specific inter-relationship between the three deformities in non-ambulatory neuromuscular patients. It can also be used, however, to assess values in these patients while they are still in the ambulatory phase of their disorder and also if they lose the ability to sit and their maximal functional position (without bracing) is supine or semi-supine. The tabular and numerical and grade charting mechanisms were established using data reviewed retrospectively to better document the relationships we were noting. Now that the documentation approach is established, prospective studies will better define the nature of the inter-relationships. Either tabular approaches (Table 1, Fig. 2a–c) or charting approaches (Fig. 3 a–f) can be used, although the complete approach is to document the numerical values in the tables at each assessment and then plot on the charts for maximum visual identification of trends. One can use either the numerical value chart or the grade value chart; the latter were created since many clinics prefer to document patients by grading systems. A separate tabular sheet would be used for each deformity at each visit but a single chart is used for angular/coverage values and another for the grade approach for visual display of deformation, flexibility/rigidity, and inter-relationships. The orthopedic surgeon and multidisciplinary clinic following these patients determine the frequency of radiological assessments and this work does not imply that each of the many parameters needs to be assessed at each visit. The establishment of a profile of investigations, however, will enable this documentation approach to outline developing deformation and relationships at these three regions beginning in their earliest stages. This categorization of the triad of deformities is applicable to all neuromuscular diagnoses and severities; outlines maximal functional level, deformity status, progression and flexibility/rigidity at each of the three sites; and shows the close relationship between spine, pelvic, and hip deformation.

Systematic documentation with charting is used widely by pediatricians for heights and weights and by pediatric orthopedic surgeons for deformities such as lower extremity length discrepancies, but for most other deformities there are few widely practiced standardized approaches. Tabular and visual (charting) documentation are valuable, however, for deformities that develop over an extended period of time, have differing patterns, and inter-relate in a causative way with other deformities.

### Deformity progression in neuromuscular patients

#### Overview

The underlying causes of the three inter-related deformities differ significantly depending on the neuromuscular disorder involved even though there are similarities in the clinical and radiographic appearances. The triadic complex is initiated by asymmetric muscle function due to either weakness or spasticity or both. Secondary deformity then worsens as irregular growth of soft tissues and the skeleton is superimposed on the abnormal positions. In general, the earlier the triad develops the worse the deformity, since growth abnormality is superimposed on positional deformity and rigidity increases with time.

Deformity in each component tends to develop early in the first decade (ages 2–5) in severe quadriparetic CP, SMA type II, the structural congenital myopathies and congenital muscular dystrophy (especially if merosin-negative). Deformity develops in DMD most rapidly in the second decade after loss of walking, especially in the absence of steroid management [13]. Steroids have been shown to prolong ambulation and decrease scoliosis in DMD [14]. The almost invariable early onset and progression of thoracolumbar scoliosis in severely involved neuromuscular patients has been well documented [2, 15–17]. The scoliosis deformity worsens with the severity of the underlying disorder, as demonstrated in Duchenne muscular dystrophy [13, 18], cerebral palsy [2, 19], and spinal muscular atrophy [17]. When patients are non-ambulatory, the severe neuromuscular scoliosis, unlike the patterns seen in ambulatory idiopathic scoliosis patients, almost always extends to involve the lumbosacral region where it contributes to pelvic obliquity [1–3, 5–8, 15–18]. Further evidence of the inter-related nature of the three sites of deformity is provided by specific studies on hip subluxation and dislocation in relation to more proximal scoliosis and pelvic obliquity in cerebral palsy [19–22], spinal muscular atrophy [21, 23, 24], and Duchenne muscular dystrophy [3, 18, 21].

#### Pathogenesis of the inter-related triad of neuromuscular deformities

In the severe neuromuscular disorders of early childhood, the lumbar and thoracolumbar scoliosis that commonly develops almost invariably includes and extends beyond the L5 vertebra into the sacrum. A hypothesis of the sequence of events is that the scoliosis leads to an associated pelvic tilt or obliquity, since there is no space for a compensatory scoliosis correction at the lowermost spinal levels. As the pelvis tilts, the hip on the high side begins to sublux, although initially in a relative sense since it is actually the acetabulum that tilts away from its normal coverage of the femoral head. The entire pelvis tilts with the low side adjacent to the convexity and the high side to the concavity of the scoliosis curve. The hip (femoral head) shows improved coverage on the low side but progressive subluxation deformity on the high side. The femoral head and neck are already positioned into coxa valga and anteversion due to non-ambulation and they further adduct relative to the malpositioned acetabulum. The normal proximal femoral development into varus is slowed in non-ambulatory patients where a coxa valga develops with the neck-shaft angle remaining high and often approaching 180° on the antero-posterior hip radiograph (Fig. 1b). Persisting femoral anteversion contributes to this radiographic finding of coxa valga. The proximal femoral deformities of coxa valga and increased anteversion combined with acetabular subluxation due to the pelvic obliquity progressively predispose to femoral head subluxation and dislocation; in addition, there is lateral acetabular under-development due to asymmetric pressure of the malpositioned head against the lateral acetabular cartilage mass, further minimizing its depth and limiting head coverage. The triad of deformities regardless of cause then follows the classic pattern of orthopedic deformity, being flexible early in the developmental sequence (passively correctable), then partially rigid and eventually markedly rigid (minimal to no change in supine position or with manual manipulations) due to soft tissue contractures and bony deformity. This documentation approach allows for awareness of the simultaneous development of these three inter-related regional structural deformities. The pathoanatomic changes of the femoral–acetabular complex are demonstrated in Fig. 1b.

#### Progression of deformities at multiple sites

We had noted previously the essentially invariable scoliosis in non-ambulatory neuromuscular patients to be lumbar or thoracolumbar, to extend and be continuous into the sacrum, and to be associated with a pelvic obliquity. In a subset of 116 patients with scoliosis, the incidence of pelvic obliquity was 98 %.

In then assessing the patterns of inter-related deformity, variability can be seen in the relative progression of deformity in the entire group of patients. Spinal deformities (scoliosis) developed more rapidly than hip deformities in 44 %, spine and hip deformities developed at the same time in 40 %, and hip deformities developed more rapidly than spine deformities in only 16 %. This variability was assessed statistically using Pearson’s chi-squared test where *p* = 0.0501, an almost significant valuation. In the latter group (hip > spine), 7 of the 10 patients were in the CP group (Table 2). We feel that an adducted hip alone in a CP patient has little tendency, in a mechanical sense, to tilt the pelvis or spine above. It is in the severely quadriparetic CP patients with global involvement that the triad of deformities invariably occurs. Some non-ambulatory cerebral palsy patients with more regional involvement (as distinct from those with global spastic quadriparesis) show hip deformity developing a considerable period of time before pelvis and spine deformity and consequently hip displacement is not always at the high pelvic side. Hodgkinson et al., in a study of 234 non-ambulatory patients with cerebral palsy, clearly recognized the triad of deformities but could not find a relationship between the side of the pelvic obliquity and the side of the scoliosis convexity or hip displacement. They found, however, that pelvic obliquity was a risk factor for hip displacement and that eventually those deformities were a direct risk factor for scoliosis [2]. In the weakest SMA patients (type I and the most involved type II) both hips may sublux and dislocate very early, even before an eventual marked pelvic obliquity establishes itself. This variability in the entire series is seen in the finding of three temporal groups of progression and the variability in the *p*-value (*p* = 0.0501, almost significant) (Table 2).

### Flexibility/rigidity documentation in pelvic obliquity

We described the methods for assessing flexibility/rigidity of spinal and hip deformity that are well accepted in current clinical practice. Pelvic flexibility decreasing obliquity can be seen on a sitting antero-posterior spinal radiograph in brace, compared with a sitting radiograph out of brace, and it is also visible at times on supine radiographs qualitatively compared to the upright sitting views. While pelvic obliquity and early flexibility are recognized in the scoliosis literature, the obliquity frequently becomes rigid and its correction is one of the main reasons why spinal fusions are extended to the pelvis [1, 7, 8, 16, 25]. Documentation of pelvic flexibility, however, unlike with spine and hip, is not standardized and generally is not done. In reality, it is usually difficult and sometimes not possible to visualize both iliac crests in neuromuscular scoliosis patients in the recommended sitting antero-posterior radiograph due to overlying braces, osteopenic bone, and pelvic rotation. In an effort to improve and standardize the documentation of flexibility of pelvic obliquity, we demonstrate a measurement in the supine position by specifically positioning the head, trunk, and pelvis along the midline of the radiological table with the lower extremities parallel to each other and to the long axis of the table, with the radiograph centered on the pelvis and showing (at a minimum) the lumbar spine, the complete pelvis, and the hips and proximal half of the femurs. This approach basically mirrors the anatomical positioning of the lower extremities and pelvis in the standing position, but with the effects of gravity and weakness removed. Further prospective studies will be needed to establish firmer guidelines, but the method appears promising and is clearly important to the concept of this documentation approach.

### Value of deformity documentation

Clinical awareness of the inter-related triad of deformities is important and charting of its development can be valuable. The presence, progression, and flexibility of these deformities warrant the improved methods of documentation defined. This is particularly true as groups of surgeons concentrate exclusively on treatment of spine or hip deformities. Treatment approaches can differ (often markedly) depending on etiology and must be based not on deformity values alone but also in relation to the specific underlying neuromuscular disorder. While the triad of deformities in the non-ambulatory neuromuscular patient invariably occurs, the variable temporal patterns of development appear to have some dependency on the specific disorder. The tabular and charting approach to the inter-related triadic deformity documentation will be increasingly helpful not only for management decisions but also as a clinical research tool.
